# Enhancement of Arsenic Trioxide-Mediated Changes in Human Induced Pluripotent Stem Cells (IPS) 

**DOI:** 10.3390/ijerph110707524

**Published:** 2014-07-22

**Authors:** Barbara Graham, Jacqueline Stevens, Phatia Wells, Jennifer Sims, Christian Rogers, Sophia S. Leggett, Stephen Ekunwe, Kenneth Ndebele

**Affiliations:** 1 Laboratory of Cancer Biology and Target Validation, Department of Biology, Jackson State University, Jackson, MS 39217, USA; E-Mails: pjwells27@yahoo.com (P.W.); jen_nsims@yahoo.com (J.S.); 2 RCMI Molecular Core Lab, Department of Biology, Jackson State University, Jackson, MS 39217, USA; E-Mail: Jacqueline.stevens@jsums.edu; 3 Department of Behavioral and Environmental Health, Jackson State University, Jackson, MS 39217, USA; E-Mail: Sophia.s.leggett@jsums.edu; 4 Department of Biology, Jackson State University, Jackson, MS 39217, USA; E-Mails: Christian.s.rogers@jsums.edu (C.R.); Stephen.ekunwe@jsums.edu (S.E.)

**Keywords:** arsenic, induced pluripotent stem cells, genotoxicity

## Abstract

Induced pluripotent stem cells (IPS) are an artificially derived type of pluripotent stem cell, showing many of the same characteristics as natural pluripotent stem cells. IPS are a hopeful therapeutic model; however there is a critical need to determine their response to environmental toxins. Effects of arsenic on cells have been studied extensively; however, its effect on IPS is yet to be elucidated. Arsenic trioxide (ATO) has been shown to inhibit cell proliferation, induce apoptosis and genotoxicity in many cells. Based on ATOs action in other cells, we hypothesize that it will induce alterations in morphology, inhibit cell viability and induce a genotoxic effect on IPS. Cells were treated for 24 hours with ATO (0–9 µg/mL). Cell morphology, viability and DNA damage were documented. Results indicated sufficient changes in morphology of cell colonies mainly in cell ability to maintain grouping and ability to remain adherent. Cell viability decreased in a dose dependent manner. There were significant increases in tail length and moment as well as destruction of intact DNA as concentration increased. Exposure to ATO resulted in a reproducible dose dependent sequence of events marked by changes in morphology, decrease of cell viability, and induction of genotoxicity in IPS.

## 1. Introduction

Arsenic is a naturally occurring element widely distributed throughout the environment, found in rocks, soil, water, air, plants and animals [1,2]. The inorganic form of arsenic is highly toxic (combined with oxygen, iron, chlorine, and sulfur) while the organic form is not thought to be linked to cancer [3]. Based on epidemiological evidence, arsenic has been listed as a human carcinogen [4,5,6]. Humans are exposed to inorganic compounds via inhalation, ingestion of food, contaminated drinking water (the major exposure route) [7] and eye or dermal contact [8]. Chronic exposure to arsenic has been associated with different types of cancer and provoking formation of various solid tumors– lung, skin, liver, bladder [9,10,11,12,13,14], renal, [15] and prostate [16] cancers—as well as other malignancies including hypertension, type 2 diabetes [17], and blackfoot disease [18]. Arsenic compounds, alone or in combination with other agents, have also been used as a therapeutic agent [19] for human disease (acute promyelocytic leukemia) as a first-line therapy resulting in high rates of complete and molecular remission [20,21] as well as agricultural applications (insecticides and fertilizers) [22,23] and poultry [24,25] and swine [26] feed. In mammalian cells, various studies have found that arsenic has a cytotoxic [27,28,29] and/or genotoxic potential [30,31,32,33,34,35,36]. A major mechanism by which arsenic exhibits its effects on target cells is through generation of reactive oxygen species (ROS) [37], loss of mitochondrial membrane potential, and release of cytochrome *c*, resulting in programmed cell death (apoptosis) [38,39,40,41]. Studies show that there is a clear induction of genotoxic effects and a decrease in the proliferation index that reflects its toxic potential [42,43]. Elucidation of the precise molecular mechanisms of arsenic’s mode of operation is critical to our understanding of how it wields its toxicity in different cells.

The great concern to the well-being and health security of humans has made arsenic a target for extensive study. Because of arsenics potential use as a therapeutic agent, it was important to determine its effect on a relatively new line of cells known as human induced pluripotent stem cells (IPS). IPS is not to be confused with embryonic stem cells (ES). ES cells and IPS cells are similar in their functions; however, they harbor subtle differences such as distinct origins and modes of derivation [44]. IPS cells have the key features of ES cells, in that they have the ability to propagate in culture indefinitely and the capacity to generate cells from all three embryonic germ layers [45,46,47].

Both play a major role in research individually as well as complementary. The discovery and isolation of stem cells brought with it the potential to understand early human development, tissue formation and differentiation through *in vitro*. As IPS become more prominent in research, it is expected that discoveries made using these cells will enhance future drug development or other therapeutic interventions [48].

The potential of IPS cells include drug discovery transformation by providing toxic compound identification, target validation and tool discovery [49,50,51,52,53]. ATO has been shown to inhibit cell proliferation, induce apoptosis and genotoxicity in many cells. The aim of this study is to determine the role ATO has on cell morphology, growth and DNA changes on IPS cells. 

## 2. Experimental Section

### 2.1. Chemicals, Reagents and Supplies

The following reagents and supplies were used: Matrigel™ (354230, BD Biosciences^®^, San Jose, CA, USA), mTeSR™1 Medium (05850, Stem Cell Technologies, Vancouver, BC, Canada), DMEM/-F-12 Medium (11330-057, Invitrogen, city, Grand Island, NY, USA), Dispase (17105-041, Invitrogen), Comet assay kit (Trevigen Inc., Gaithersburg, MD, USA). Arsenic trioxide (ATO, 1,000 ppm, SA449-100), 6-well plates (140675, Nunc), and sterile glass serological pipettes (13-678-27E, Fisher) were purchased from Thermo Fisher Scientific (Suwanee, GA, USA). All other chemicals (analytical reagent grade) were purchased from commercial sources. 

### 2.2. Cell Line

IPS cells, (Foreskin)1-MCB-01, were purchased from University of Wisconsin (Madison, WI, USA) Laboratory of Dr. James Thomson through the supporting organization of WiCell Research Institute (Madison, WI, USA).

### 2.3. Cell Culture and Exposure

Protocol for plating cells were followed based on WiCell Feeder Independent Pluripotent Stem Cell Protocols (SOP Number: SOP-SH-002). Briefly, matrigel plates were prepared at least 2 hours before cell culturing (0.5 mg/6 well plate) and matrigel removed immediately before adding cell suspension. Cells were suspended in 3 ml mTeSR™1 Medium. To the 6-well plates, 1.5 mL mTeSR™1 medium and 0.5 mL of the cell suspension was added drop-wise into each well. Plates were placed gently into 37 °C in 5% CO_2_ humidified incubators. Media was changed daily. Cells were passaged using 1 mL of room temperature filtered sterile dispase solution (dispase 2 mg/mL DMEM-F12 Medium). Plate(s) was incubated for 3 minutes, and viewed under microscope to determine if cells were partially detached from plate. Cells were gently washed two times with 1 mL of DMEM/F-12, followed by cell scaping using a 5 mL glass pipette containing 1 mL of medium. Contents were pooled into a sterile conical tube and gently pipetted to dislodge any colonies. Cells were resuspended to make a total of 2.0 mL of medium and cells in each of the new wells (0.5 mL of cell suspension + 1.5 mL of mTeSRTM1 medium). Cells were allowed to grow until signs of differentiation were noticed (day 4), Cell colonies were counted in each well of the 6 plates to ensure consistency in plating. Each of the six plates represented a concentration of arsenic. A stock solution of ATO (100 µg /mL) was prepared and diluted to appropriate concentrations in cell culture medium (mTeSR™1). The plates were treated with ATO at concentrations of 0, 1, 3, 5, 7 and 9 µg/mL for 24 hours. 

### 2.4. Cell Morphology

After the 24 hour incubation period with arsenic, morphology of IPS was observed using an Olympus Inverted Phase Contrast Microscope with Camera (C-Squared, magnification 200×) and photographs of each well was taken.

### 2.5. Cell Viability/Cytotoxicity

After exposure to arsenic, cell colonies were counted in each well and compared with the control using the phase-contrast microscope. Untreated sets (0) were used as the controls. To demonstrate the growth inhibition induced in induced pluripotent stem cells by arsenic after 24 hours, viable cell numbers were also counted using trypan blue staining.

### 2.6. Determination of DNA Damage (Genotoxicity)

Comet Assay was used to evaluate genotoxicity by quantifying and analyzing DNA damage in individual cells. The assay was performed according to the instructions of the manufacturer (Trevigen, Inc., Gaithersburg, MD, USA)) with slight modifications. Cells were incubated for 24 hours in 5% CO_2_ at 37 °C in the presence of ATO. After incubation, cells were detached using dispase, centrifuged, washed three times with cold PBS and viability evaluated using the trypan blue exclusion assay. The pellet was re-suspended (1 × 10^5^cells/mL) in PBS (Ca^2+^ and Mg^2+^ free). The cells were combined with molten LMAgarose (37 °C) at a ratio of 1:10 (v/v), and 75 μL was immediately pipetted onto CometSlide^TM^. The slides were placed flat in a refrigerator at 4 °C for 10–20 min, and then immersed in prechilled lysis solution at 4 °C for 45 minutes. Excess buffer was drained from slides and they were immersed in alkaline Unwinding Solution for 60 minutes in the dark at room temperature. Slides were placed in electrophoresis tank and covered with 950 mL prechilled alkaline electrophoresis solution, with power supply set at 21 V for 30 minutes. Excess electrophoresis solution was drained, slides were immersed twice in dH_2_O for 5 minutes each, then in 70% ethanol for 5 minutes. Samples were then allowed to dry overnight at room temperature, stained with SYBR Green and allowed to set for 24 h. For examining stained comet slides, 150 comets were scored per concentration and 75 comets were randomly selected and viewed using the Olympus Epifluorescence Microscope, and analyzed by the LAI Automated Comet Assay Analysis System (Loates Associates, Inc., Westminister, MD, USA). The parameters (tail length and olive tail moment) were selected for DNA damage quantification in the IPS as determined by the software.

### 2.7. Statistical Analysis

A minimum of three independent experiments were carried out in duplicate for each experiment. Data was expressed as the mean (±standard deviation). Student’s paired t-test was used to analyze the difference between the control and ATO-treated cells. All *p*-values <0.05 were considered to be significant. QI Macros software was used. 

## 3. Results

### 3.1. Effect of Arsenic Trioxide on Morphological Changes

**Figure 1:**
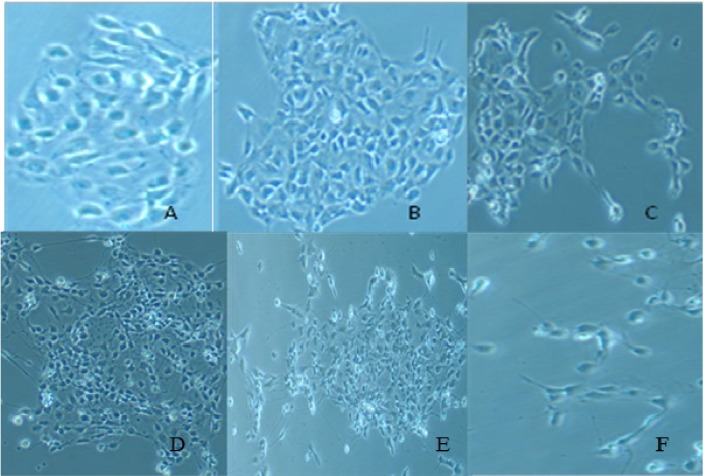
Morphological changes in IPS colonies after 24 hour ATO exposure. Control (**A**) and ATO-treated (**B**) 1.0, (**C**) 3.0, (**D**) 5.0, (**E**) 7.0 and (**F**) 9.0 μg/mL IPS were observed using an Olympus Inverted Phase Contrast Microscope with Camera (C-Squared, mag 200×). A and B maintained their original shape and continued to grow in a uniform manner, while at (**C**) 3 µg/mL cells begin to lose adherence to plate and become disengaged. At the higher concentrations 5−9 µg/mL (D−F), cells lost all ability to remain attached and lost the round shape associated with IPS.

Comparison of the morphology of control/untreated and ATO-treated IPS cells was observed using an Olympus Inverted Phase Contrast Microscope with Camera (C-Squared, mag 200×) at concentrations of 0, 1, 3, 5, 7, and 9 µg/mL (Figure 1A−F). After 24 hour ATO exposure, changes in the morphology were visible in cells with 3 μg/mL and above (Figure 1C−F). Cells began to detach from the surface of the plate, and lose their round shape adopting a more spherical one. Cells were no longer in a uniform state being held together in colonies. As concentration of arsenic increased (5, 7, and 9 µg/mL), the cell colonies began to disaggregate into single cells (Figure 1D−F). 

### 3.2. Effect of Arsenic Trioxide on Cell Viability

**Figure 2:**
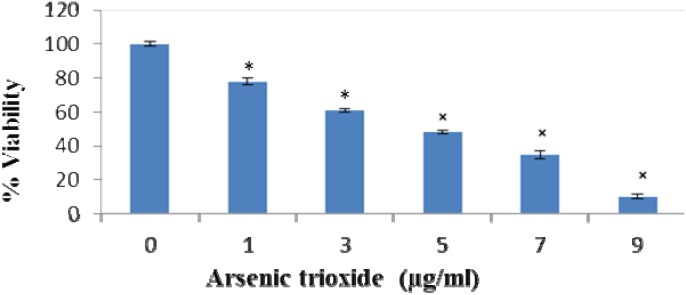
Cell viability of ATO-treated IPS after 24 hour exposure. IPS were treated for treated for 24 hours with ATO (0, 1, 3, 5, 7 and 9 µg/mL). Results showed a dose dependent decrease in cell viability with the a LD_50_ value being ~4.5 μg/mL. Each value represents the mean ± standard error of three experiments performed in triplicate. Student’s paired t-test was used to analyze the difference between the control and ATO-treated cells. All *p*-values <0.05 were considered to be significant denoted by *.

We also quantified the extent of cell viability of human IPS in the presence of ATO using Trypan Blue Exclusion Assay. Light microscopy was used to distinguish viable from non-viable cells. The results demonstrated a concentration-dependent cytotoxicity after exposure to arsenic (Figure 2). The results observed after 24 h of exposure to ATO (0, 1, 3, 5, 7 and 9 µg/mL) were 100%, 78%, 62.0%, 45%, 37% and 12% respectively, showing a LD_50_ value of ~4.5 μg/mL. 

### 3.3. Arsenic Trioxide Promotes DNA Damage

**Figure 3:**
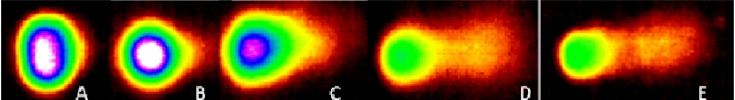
Representative microphotographs of Comets indicating changes in DNA content based on increasing ATO concentration after 24h exposure. (**A**) 0, (**B**) 1, (**C**) 3, (**D**) 5, and (**E**) (7 μg/mL ATO) represent damaged DNA , results showed increased damage as indicative of the increase in tail length.

**Figure 4:**
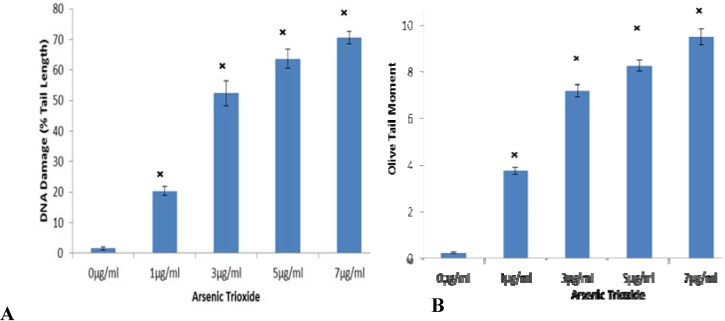
Tail Length and Olive tail moment after 24 hour of ATO exposure to IPS cells. (**A**) The tail length increased as dose increased showing an increase in DNA damage. (**B**) Olive tail moment which shows the smallest detectable size of migrating DNA (reflected in tail length), these results were consistent with tail length and the comet representative. All *p*-values <0.05 were considered to be significant as denoted by ^×^.

To examine whether ATO induces DNA damage, we performed a comet assay, which can be used to detect single cell DNA damage by the cellular elution patterns in agarose gels. The comet assay showed that ATO altered the elution profiles by promoting the accumulation of DNA damage. Comparison of DNA damage for controls (0) and ATO treated cells was measured as percent tail DNA and olive tail moment. The microphotographs (Figure 3A−E) represent changes due to damaged DNA at 0, 1, 3, 5, and 7 μg/mL, respectively after 24 h ATO exposure. The cells exposed to increasing concentrations of ATO showed more DNA damage in cells than the control cells. At the highest concentration (9 μg/mL), cells were too disintegrated for analysis, therefore, are not shown (Figure 3). In the Comet assay, the tail length measure the distance of DNA migration from the body of the nuclear core after 24 h of exposure to different concentrations of ATO. Results showed an increase in the extent (tail length) of DNa damage as concentrations increased (Figure 4A). Olive tail moment shows the smallest detectable size of migrating DNA (reflected in the comet tail length) and the number of relaxed/broken pieces (represented by the intensity of DNA in the tail) was recorded. Results were consistent with tail length percent (Figure 4B). 

## 4. Discussion

Epidemiologic studies have demonstrated that a close association exists between the elevated levels of arsenic and the incidence of certain cancers. Arsenic has been categorized as a human carcinogen based on its cytotoxic effects. There are several reports on the effect of arsenic on certain cell lines but its action on the newly derived induced pluripotent stem cells has yet to be discovered. In this study, our team reports on the effect that ATO has on morphology, proliferation/survival and genotoxicity of human induced pluripotent stem cells. This study is very important to the scientific world because scientist can focus on the potential use of IPS as a tool for drug development, transplantation medicine and other therapeutic modeling [48]. 

Arsenic has been shown to induce changes in cell morphology [54,55,56]; therefore, we first examined morphological changes that ATO causes on IPS. As studies have shown in other cell lines, changes in cell colony integrity and adherence occurred when IPS was treated with ATO. Cell shrinkage occurred in all treated cells but was most prominent at higher concentrations. Cells were no longer able to maintain adherence as levels increased above 3 µg/mL. Once the cells lost adherence ability, survival decreased. Our results, as does existing literature, demonstrated that ATO induce morphological changes including inability to maintain a round shape and cells begin to scatter having no definite shape, this in turn affected cells ability to maintain its consistent shape and adherence pattern. 

The growth and survival of many different cells have been markedly inhibited by arsenic at low levels of treatment [27,35,40,43,57,58,59,60]. In this study, cell proliferation and survival were assessed using the trypan blue exclusion assay. Results showed that arsenic inhibited cell viability in a dose dependent manner when exposed to human induced pluripotent stem cells which is consistent with previous studies suggesting that the arsenic decreases cell viability in a dose dependent manner. 

Arsenic trioxide’s effect on genotoxicity has been analyzed extensively in a wide range of *in vitro* studies [29,35,36,43]. Overall, studies have shown that it is likely that arsenic directly and/or indirectly induces genotoxic effects, including an increase in micronucleus frequency and a decrease in the proliferation index that reflects its toxic potential. In use of the Comet assay, studies have shown that ATO treatment increased accumulation of DNA damage [34,35,61]. In the present study, various concentrations of ATO caused an increase in tail length and olive tail moment which was indicative of DNA damage after 24 hours of treatment when compared with controls.

Arsenic is a chemotherapy agent, its mode of action is not completely understood but it has been shown to block the growth of some cancer cells. The dosage use in humans is based on body weight and range from 0.06 to 0.2 mg/kg/day [62]. Because this is the first published study using IPS and ATO, we used concentrations of ATO that have been shown to cause an effect *in vitro.* Further studies will be done to look at different time periods and dosage. 

## 5. Conclusions

In conclusion, ATO cause significant changes in induced pluripotent stem cells. Of interest for future investigations will be the long-term effect of arsenic at very low levels on this particular cell line as well as alterations in gene expression. Our results demonstrate ATO enhanced changes in morphology by decreasing adherence and cells lost cell membrane integrity allowing for non-conformity; cell viability decreased as concentration increased; and an induction of DNA strand breaks in human induced pluripotent stem cells. Exposure to arsenic may change the way cells communicate with each other, as well as alter their functionality. Although much additional research is needed, this is a major step toward identifying some of the changes afforded human induced pluripotent stem cells by arsenic. As more information is reported on arsenic, it is important to broaden the knowledge of its human health effects both short term and long term. 
